# Efficacy and safety of secukinumab in patients with giant cell arteritis: study protocol for a randomized, parallel group, double-blind, placebo-controlled phase II trial

**DOI:** 10.1186/s13063-021-05520-1

**Published:** 2021-08-17

**Authors:** Nils Venhoff, Wolfgang A. Schmidt, Peter Lamprecht, Hans-Peter Tony, Christine App, Christian Sieder, Carolin Legeler, Claudia Jentzsch, Jens Thiel

**Affiliations:** 1 Department Innere Medizin, Klinik für Rheumatologie und Klinische Immunologie, Vaskulitiszentrum Freiburg, Universitätsklinikum Freiburg, Hugstetterstrasse 55, D-79106 Freiburg, Germany; 2 Immanuel Krankenhaus Berlin, Klinik für Innere Medizin, Abteilung Rheumatologie und Klinische Immunologie in Berlin-Buch, Lindenberger Weg 19, D-13125 Berlin, Germany; 3 Universität zu Lübeck, Klinik für Rheumatologie und klinische Immunologie, Ratzeburger Allee 160, D-23538 Lübeck, Germany; 4 Medizinische Klinik II, Universitätsklinik, Rheumatology/Immunology, Oberduerrbacher Strasse 6, D-97080 Wuerzburg, Germany; 5 Department of Immunology, Hepatology & Dermatology, Novartis Pharma GmbH, Roonstrasse 25, D-90429 Nuremberg, Germany; 6 Novartis Pharma AG, Basel, Switzerland

**Keywords:** Giant cell arteritis, Secukinumab, Phase II trial, Placebo, Double-blind

## Abstract

**Background:**

One key pathological finding in giant cell arteritis (GCA) is the presence of interferon-gamma and interleukin (IL)-17 producing T helper (Th) 1 and Th17 cells in affected arteries. There is anecdotal evidence of successful induction and maintenance of remission with the monoclonal anti-IL-17A antibody secukinumab. Inhibition of IL-17A could therefore represent a potential new therapeutic option for the treatment of GCA.

**Methods:**

This is a randomized, parallel-group, double-blind, placebo-controlled, multi-center, phase II study in which patients, treating physicians, and the associated clinical staff as well as the sponsor clinical team are blinded. It is designed to evaluate efficacy and safety of secukinumab compared to placebo in combination with an open-label prednisolone taper regimen. Patients included are naïve to biological therapy and have newly diagnosed or relapsing GCA. Fifty patients are randomly assigned in a 1:1 ratio to receive either 300 mg secukinumab or placebo subcutaneously at baseline, weeks 1, 2 and 3, and every 4 weeks from week 4. Patients in both treatment arms receive a 26-week prednisolone taper regimen. The study consists of a maximum 6-week screening period, a 52-week treatment period (including the 26-week tapering), and an 8-week safety follow-up, with primary and secondary endpoint assessments at week 28. Patients who do not achieve remission by week 12 experience a flare after remission or cannot adhere to the prednisolone tapering will enter the escape arm and receive prednisolone at a dose determined by the investigator’s clinical judgment. The blinded treatment is continued. Two optional imaging sub-studies are included (ultrasound and contrast-media enhanced magnetic resonance angiography [MRA]) to assess vessel wall inflammation and occlusion before and after treatment. The primary endpoint is the proportion of patients in sustained remission until week 28 in the secukinumab group compared to the proportion of patients in the placebo group. A Bayesian approach is applied.

**Discussion:**

The trial design allows the first placebo-controlled data collection on the efficacy and safety of secukinumab in patients with GCA.

**Trial registration:**

ClinicalTrials.gov NCT03765788. Registration on 5 December 2018, prospective registration, EudraCT number 2018-002610-12; clinical trial protocol number CAIN457ADE11C.

## Background

Giant cell arteritis (GCA) is a systemic large vessel vasculitis affecting people aged 50 years and older. The two main types of large vessel vasculitis are Takayasu arteritis (TA) and GCA. Large vessel vasculitis covers the spectrum of primary vasculitis which leads to chronic granulomatous inflammation of larger arteries, e.g., temporal arteries, the aorta, or its major branches [1]. Up to 60% of patients with GCA also show features of polymyalgia rheumatica (PMR) which are overlapping inflammatory rheumatic disorders. Clinical signs and symptoms of PMR include stiffness and aching in the shoulder and pelvic girdles and cervical region. Conversely, 16–21% of patients with PMR have GCA [2]. GCA is the most common vasculitis in adulthood. Persons in Northern Europe hold the highest incidence of GCA and PMR, particularly persons of Scandinavian descent [3].The incidence of GCA in the USA is 18 per 100,000 which is the most frequent primary vasculitis. According to estimates, the number of GCA diagnoses will exceed 3 million cases by 2050 leaving approximately 500,000 people visually impaired [4]. Typical clinical manifestations of GCA related to the inflammation of large- and medium-sized arteries are new-onset headaches, jaw claudication (cramping pain and/or fatigue felt in the jaw muscles during mastication), scalp tenderness, and visual disturbances. Characteristic systemic manifestations include fever, malaise, weight loss, and polymyalgia [5]. The most feared complication of GCA is irreversible, permanent visual loss representing a severe medical emergency. Therefore, prompt and effective immunosuppressive treatment is crucial in GCA [6, 7].

High-dose glucocorticoids are still the standard of care therapy and effectively reduce vascular inflammation [8, 9]. However, this treatment has serious disadvantages for the patients: relapses and treatment failures are common, and more than 80% of patients suffer from serious adverse events (SAE) [8, 9]. In addition, many patients have relative contraindications to glucocorticoid therapy. Thus, there is an unmet need for glucocorticoid-sparing agents, which allow for long-term remissions in the absence of those adverse effects associated with glucocorticoid treatment. Anti-tumor necrosis factor (TNF) inhibitors, azathioprine, and methotrexate could serve as potential alternatives, but treatment results are not conclusive [9–11]. Promising results came from the GiACTA-trial [12, 13]. In that trial, interference of interleukin-6 (IL-6) signaling with tocilizumab, an IL-6 receptor antagonist, had a beneficial effect in patients with GCA, which eventually led to the approval of tocilizumab for GCA. However, tocilizumab suppresses acute phase reactants, which are integral to currently used remission and relapse criteria. Reichenbach et al. analyzed magnetic resonance angiography (MRA) vessel wall signs from a randomized controlled trial of tocilizumab to treat GCA, which showed normalization of MRA signals of only one third of patients after 52 weeks. One-third of patients showed persistent or increased late vessel wall enhancement [14]. It remains unclear whether these findings are of prognostic importance.

Thus, there is a need for further glucocorticoid-sparing treatment alternatives other than tocilizumab. This phase II trial investigates the efficacy and safety of secukinumab, a fully human monoclonal antibody that selectively inhibits IL-17A, in patients with active GCA. Secukinumab received approval for adult treatment of moderate to severe plaque psoriasis, active psoriatic arthritis, active non-radiographic axial spondyloarthritis, and active ankylosing spondylitis in numerous countries, including the EU and the USA [15].

## Design and methods

### Rationale for study design

The herein presented randomized, parallel-group, double-blind, placebo-controlled trial design is closely aligned with the design of phase II trials during the clinical development program for secukinumab for other indications (plaque psoriasis, ankylosing spondylitis, non-radiographic axial spondyloarthritis, psoriatic arthritis), in which secukinumab demonstrated efficacy and safety.

Patients with newly diagnosed or relapsing GCA who are naïve to biological therapy and already receive glucocorticoids with a prednisolone equivalent dose of 25–60 mg/day are considered for assessing efficacy of secukinumab compared with placebo in GCA.

The rationale for using IL-17A as therapeutic target and the rationale for the choice of glucocorticoid combination therapy are highlighted in the Appendix.

### Design

**Fig. 1:**
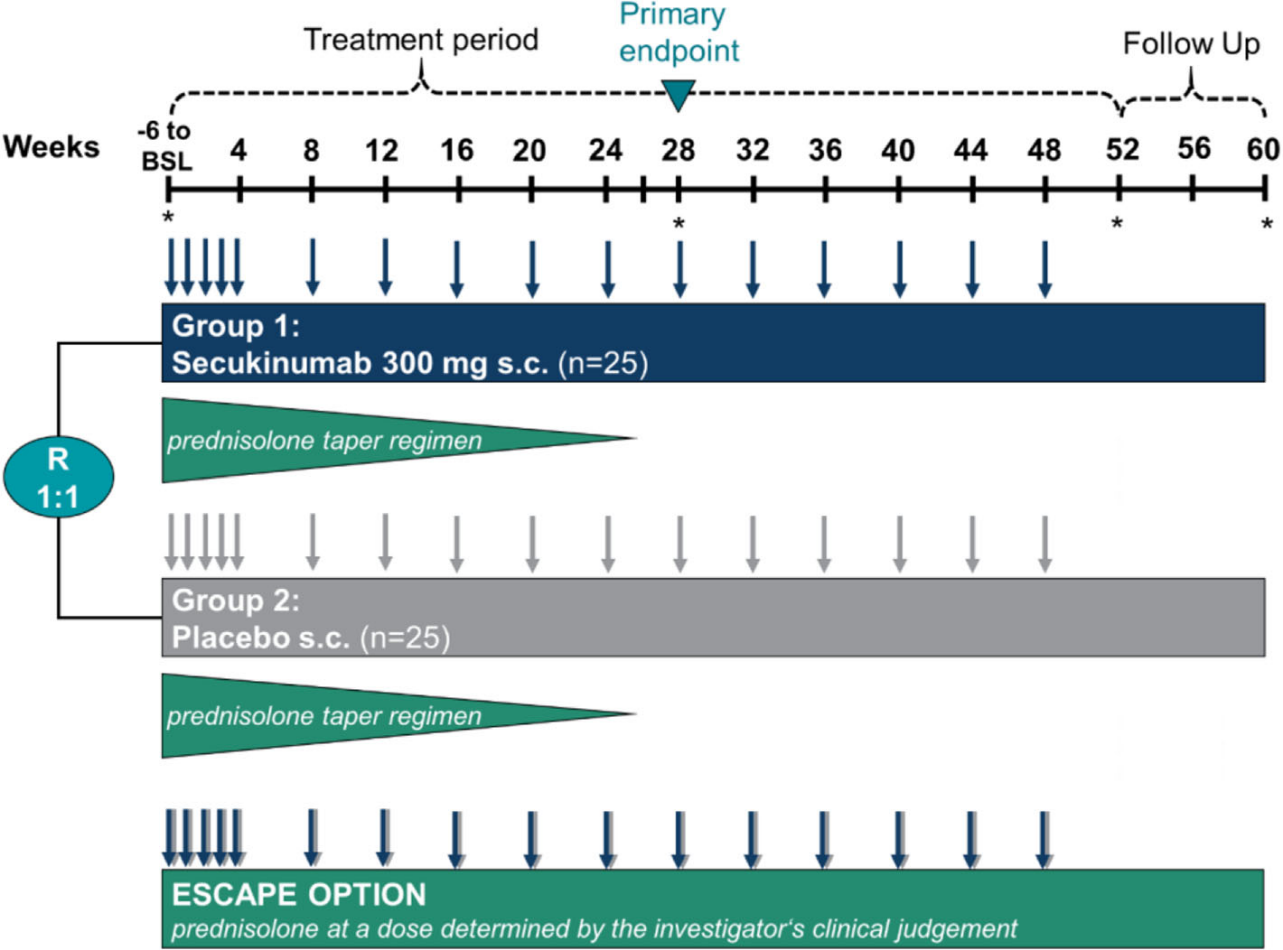
Study design. Arrows indicate the application time points of secukinumab and placebo; stars indicate the time points at which imaging (ultrasound and/or magnetic resonance angiography) is performed. BSL, baseline; mg, milligram; s.c., subcutaneous; R, randomization (randomization occurs after the 6-week screening period at baseline); n, number

As shown in Fig. 1, the trial is set up as a randomized, parallel-group, double-blind, placebo-controlled, multicenter, phase II study. The goal is to evaluate the efficacy and safety of secukinumab compared to placebo in combination with an open-label 26-week prednisolone taper regimen.

Initially, during an up to 6-week screening period, patients may receive glucocorticoids for the treatment of GCA at the discretion of the investigator. By the end of this screening period, patients should be able to switch to the sponsor-provided prednisolone in order to follow the protocol-defined prednisolone tapering regimen. Then, a 52-week treatment period with the initial 26-week prednisolone taper and an 8-week safety follow-up will succeed.

Patients who do not achieve remission by week 12 experience a flare after remission or cannot adhere to the prednisolone taper regimen will enter the “escape” arm. These patients will receive prednisolone at a dose determined by the investigator based on the clinical findings. They will continue treatment with secukinumab or placebo without unblinding.

Patients are randomized to one of the following treatment arms in a 1:1 ratio:

- Group 1: Secukinumab 300 mg subcutaneous (s.c.) + 26-week prednisolone taper regimen
- Group 2: Placebo s.c. + 26-week prednisolone taper regimen

Table 1 shows an abbreviated version of the study schedule.

**Table 1:** Study schedule (selection)

Period	Screening*		BL	Treatment period															EOT	Safety-FU	
Week	−6 to BL	≤ 4 weeks from baseline	0	1	2	3	4	8	12	16	20	24	28	32	36	40	44	48	52	56	60
Inclusion/exclusion criteria	x	x	x																		
Relevant medical history/concomitant diseases	x																				
Demography	x																				
GCA medical history and previous therapies	x																				
Prior/concomitant medications/non-drug therapy	x	x	x	x	x	x	x	x	x	x	x	x	x	x	x	x	x	x	x	x	x
Administration of s.c. study treatment			x	x	x	x	x	x	x	x	x	x	x	x	x	x	x	x			
Prednisolone treatment (26-week taper)			x	x	x	x	x	x	x	x	x	x	x								
GCA assessment (signs and symptoms)	x	x	x	x	x	x	x	x	x	x	x	x	x	x	x	x	x	x	x	x	x
Ultrasound (selected sites)	x												x						x		x
MRA assessment (selected sites)	x												x						x		x
Patient reported outcomes (PGA, EQ-5D, SF-36, FACIT-fatigue)			x				x	x	x	x	x	x	x		x		x		x		
PhGA			x				x	x	x	x	x	x	x		x		x		x		
GTI			x				x	x	x	x	x	x	x		x		x		x		
ESR and CRP			x	x	x	x	x	x	x	x	x	x	x	x	x	x	x	x	x		
Pharmacokinetic assessments			x				x		x				x								x
Pharmacogenetics			x																		
Anti-secukinumab antibodies			x				x		x				x		x		x		x		x
Laboratory assessment		x	x				x		x				x		x		x		x		

*BL*, baseline (randomization occurs after the 6-week screening period at baseline); *CRP*, C-reactive protein; *EOT*, end of treatment; *ESR*, erythrocyte sedimentation rate; *FU*, follow-up; *GCA*, giant cell arteritis; *GTI*, glucocorticoid toxicity index; *MRA*, magnetic resonance angiography; *PGA*, Patient’s Global Assessment; *PhGA*, Physician’s Global Assessment

^*^Screening Visit 1 and Visit 2 can be performed on the same day if appropriate

An extension phase (24-week) was added to the core phase (28-week) in an amendment (2nd) to assess the effect of secukinumab after completed glucocorticoid tapering with regard to sustained remission, the potential steroid-sparing effect, the potential effect on vascular imaging, quality of life (QoL), safety, and tolerability up to week 52. Patients are expected to remain in the study for 52 weeks (core phase + extension phase) plus 8-week safety follow-up. Blinded secukinumab or placebo treatment will be given until week 48 for final assessments in week 52. Two follow-up visits are scheduled at weeks 56 and 60 (Fig. 1 and Table 1).

### Participants

The objective is to include 50 patients diagnosed with GCA in Germany. Eleven study centers with experience in the field of GCA were chosen for patient recruitment. Patients with new onset of GCA and relapsing GCA (both with active GCA within 6 weeks before baseline) are eligible for trial participation. Table 2 summarizes the eligibility criteria for the trial.

**Table 2:** Eligibility criteria (selection)

Inclusion criteria	Exclusion criteria
► Diagnosis of GCA classified according to the following criteria: • Age at onset of disease ≥ 50 years. • History of ESR ≥ 30 mm/h or CRP ≥ 10 mg/L. • Unequivocal cranial symptoms of GCA (new-onset localized headache, scalp or temporal artery tenderness, ischemia-related vision loss, or otherwise unexplained mouth of jaw pain upon mastication) **AND/OR** symptoms of PMR (defined as shoulder and/or hip girdle pain associated with inflammatory morning stiffness). • Temporal artery biopsy revealing features of GCA **AND/OR** evidence of large-vessel vasculitis by angiography or cross-sectional imaging study such as MRA, positron emission tomography-computed tomography (PET-CT), or ultrasound. ► Patients with new onset GCA or relapsing GCA: • Definition of new onset: diagnosis of GCA within 6 weeks prior to baseline visit. • Definition of relapsing GCA: diagnosis of GCA (in accordance with inclusion criterion no. 4) > 6 weeks prior to baseline visit and in the meantime achieved remission (absence of signs and symptoms attributable to GCA and normalization of ESR [< 30 mm/h] and CRP [< 10 mg/L]) including previous treatment with ≥ 25 mg/day prednisolone equivalent for ≥ 2 weeks. ► Active disease as defined by the presence of signs and symptoms of GCA (cranial or PMR) and elevated ESR ≥ 30 mm/h, or CRP ≥ 10 mg/L, attributed to active GCA within 6 weeks of baseline. ► Prednisolone dose of 25–60 mg/day at baseline.	► Previous exposure to secukinumab or other biologic drug directly targeting IL-17 or IL17 receptor. ► Patients treated with any cell-depleting therapies including but not limited to anti-CD20 of investigational agents (e.g., anti-CD3, anti-CD4, anti-CD5 or anti-CD19). ► Patients who have previously been treated with any biologic agent including but not limited to tocilizumab, sirukinumab, abatacept, or TNF-alpha inhibitors (infliximab, adalimumab, etanercept, certolizumab, golimumab). ► Patients who have previously been treated with tofacitinib or baricitinib. ► Patients treated with intravenous immunoglobulins or plasmapheresis within 8 weeks prior to baseline. ► Patients treated with cyclophosphamide, tacrolimus or everolimus with 6 months prior to baseline. ► Patients treated with hydrochloroquine, cyclosporine A, azathioprine, sulfasalazine, mycophenolate mofetil within 4 weeks of baseline. ► Patients treated with leflunomide within 8 weeks of baseline unless a cholestyramine washout has been performed in which case the patient must be treated within 4 weeks of baseline. ► Patients treated with an alkylating agent. ► Patients requiring systemic chronic glucocorticoid therapy for any other reason than GCA. ► Chronic systemic glucocorticoid therapy over the last 4 years or longer, or inability, in the opinion of the investigator, to withdraw glucocorticoid therapy through protocol-defined taper regimen due to suspected or established adrenal insufficiency. ► Patients requiring chronic high potency opioid analgesics for pain management. ► Active ongoing inflammatory diseases or underlying metabolic, hematologic, renal, hepatic, pulmonary, neurologic, endocrine, cardiac, infectious or gastrointestinal conditions, which in the opinion of the investigator immunocompromises the patients and/or places the patients at unacceptable risk for participation in an immunomodulatory therapy. ► History of renal trauma, glomerulonephritis, or patients with one kidney only, or a serum creatinine level exceeding 1.8 mg/dL (159.12 μmol/L). ► Screening total white blood cell count < 3000/μl, or platelets < 100 000/μl, or neutrophils < 1500/μl, or hemoglobin < 8.3 g/dL (83 g/L). ► Major ischemic event, unrelated to GCA, with 12 weeks of screening. ► Known infection with human immunodeficiency virus, hepatitis B or hepatitis C at screening or randomization. ► Life vaccination within 6 weeks prior to baseline or planned vaccination during the study participation until 12 weeks after last study treatment administration.

Screening will be up to 6 weeks in order to be able to adhere to the protocol-defined prednisolone taper regimen and to allow for sufficient time for the washout of relevant medication (e.g., leflunomide).

A list of study sites can be obtained at https://clinicaltrials.gov/ct2/show/NCT03765788.

### Study treatment

#### Secukinumab/placebo

The dose regimen, route of administration, and duration of secukinumab treatment was selected based on its proven efficacy and safety in other inflammatory diseases. Secukinumab 300 mg s.c. is the approved dose for moderate to severe plaque psoriasis and for patients with psoriatic arthritis who are anti-TNF-alpha inadequate responders or who have concomitant moderate to severe plaque psoriasis. The approved secukinumab label also covers treatment with 150 mg for psoriatic arthritis (i.e., those not meeting 300-mg dose requirements) and ankylosing spondylitis. Recently, the option to up-titrate to a dose of 300 mg was introduced based on the results from the Measure 3 study [16].

Placebo is the chosen control for this study as it is unknown whether secukinumab can improve signs and symptoms of GCA.

Patients receive secukinumab (2 × 150 mg s.c.) and placebo injections (2 injections s.c.) on site administered by site staff. The pre-filled syringes are packed in a double-blind manner and do not need to be prepared. During the loading phase, the patients receive secukinumab/placebo at baseline, weeks 1, 2, 3, and 4; thereafter, in the maintenance phase, injections are given every 4 weeks up to week 48 (last dose) at the study centers.

Interruptions of the study treatment are possible, if the investigator identifies a significant risk for the patient. Discontinuation of the study treatment does not require the patient to be discontinued from the study and the ongoing visits, except for a withdrawal of informed consent by the patient. In case of premature study discontinuation of a patient, every effort should be made to achieve the assessments that were scheduled for week 52 (see Table 2).

#### Prednisolone

The site staff dispenses open-label prednisolone with tablets of 1 mg, 5 mg, 10 mg, and 20 mg. This co-administered treatment with oral prednisolone follows a taper regimen from a dose of 25–60 mg/day at baseline to 0 mg/day at week 27. Prednisolone taper regimens are allocated depending on the patient’s prednisolone level at baseline (Table 3). From week 8 on, all patients will receive the same prednisolone level (15 mg/day) and will continue to taper down to 0 mg/day.

**Table 3:** Prednisolone tapering regimen

	60–40 mg/day at baseline	40–25 mg/day at baseline
Week	Dose mg/day	Dose mg/day
0	60–40	40–25
1	55–35	35–22
2	50–30	30–21
3	45–28	27–20
4	35–25	25–19
5	30–22	22–18
6	25–21	20–17
7	20	16
8	15	
9	13	
10	12	
11	10	
12	9	
13	8	
14	7	
15	6	
16	6	
17	5	
18	5	
19	4	
20	4	
21	3	
22	3	
23	2	
24	2	
25	1	
26	1	
27	0	
28	0	

The patients will use a diary to document prednisolone application.

### Rescue medication

This study design includes an “escape” arm for patients who do not achieve remission by week 12, experience a flare after remission or cannot adhere to the prednisolone taper regimen. They will receive prednisolone at a dose determined by the investigator’s clinical judgment and will continue to receive secukinumab or placebo in a blinded manner. Although these patients are “non-responders” for the primary endpoint, their subsequent follow-up will provide important data for the analysis of other feasible endpoints, e.g., cumulative prednisolone dose.

### Relevant concomitant care

Patients must be on a prednisolone dose of 25 to 60 mg/day at baseline in order to be included in the study.

Patients taking methotrexate (≤ 25 mg/week) at study entry are allowed to continue their medication provided they have taken it for at least 3 months and are on a stable dose for at least 4 weeks prior to randomization and throughout the study. These patients must be taking folic acid supplementation before randomization and during the study to minimize the likelihood of methotrexate associated toxicity.

Vitamin D treatment is strongly recommended during the study.

### Randomization and blinding

The study consists of a maximum 6-week screening period after which all patients who have been deemed eligible by the investigator receive the lowest available randomization number at baseline. This number assigned the patient to one of the treatment arms (1:1 ratio) while treatment assignment was unbiased and concealed from patients and study site staff. Permuted randomization blocks were applied. The randomization number corresponded to the number on the medication pack. The investigator enters the randomization number in the electronic case report forms. Additionally, the patient randomization list was produced by or under responsibility of the sponsor’s biometry department using a validated system that ensures a specified ratio. Randomization data is strictly confidential until the time of un-blinding (database lock). It will not be accessible by anyone involved in the study. The identity of treatments will be concealed by the use of study medication that is identical in packaging, labeling, schedule of administration, appearance, and odor. The sponsor of the study provides a double-blind supply of secukinumab and placebo pre-filled, blinded syringes, and open-label supply of prednisolone tablets. Un-blinding occurs in case of an emergency and at the end of the study. A separate sponsor clinical team will be un-blinded for 3 interim analyses (IA): IA1 (when 50% patients have completed W28), IA2 (when 50% of patients have completed W52), and IA3 (when 100% of patients have completed W28).

### Efficacy assessments

#### Primary objective

The primary objective is to evaluate the efficacy of secukinumab compared to placebo in combination with a 26-week prednisolone taper regimen. The primary efficacy endpoint is the proportion of patients with GCA in sustained remission at week 28. Patients are in sustained remission if they are without flare until week 28 and in adherence to the protocol prednisolone taper regimen. A flare is defined as recurrence of signs and symptoms after remission and/or ESR ≥ 30 mm/h and/or CRP ≥ 10 mg/L attributable to GCA as per investigator’s judgment.

#### Secondary objectives

The secondary objectives are to evaluate the efficacy of secukinumab versus placebo in combination with a 26-week prednisolone taper regimen in patients with GCA measured by the following:

- Remission rate at week 12.
- Time to first flare of GCA after clinical remission up to week 52.
- Total cumulative prednisolone dose up to weeks 28 and 52.
- Proportion of patients with GCA with sustained remission at week 52.
- Proportion of patients with a prednisolone dose of less than 5 mg/day at weeks 19, 28 and 52.
- Changes from baseline in disease activity and patient-reported outcome measures at weeks 4, 8, 12, 16, 20, 24, 28, 36, 44, and 52 for each of the following:
  - Physician’s Global Assessment (PhGA) visual analog scale (VAS)
  - Patient-reported outcomes (PROs):
    - Patient’s Global Assessment (PGA) VAS
    - Functional Assessment of Chronic Illness Therapy Fatigue (FACIT-Fatigue)
    - Short form 36 (SF36) (PCS and MCS scores)
    - EuroQoL-5D-5L (EQ-5D-5L)
- Changes from baseline in CRP and ESR at weeks 4, 8, 12, 16, 20, 24, 28, 36, 44, and 52.

### Exploratory assessments

#### Pharmacokinetics

Site personnel collect pharmacokinetic samples at different time points (see Table 1). A laboratory manual will outline the instructions for sample collection, numbering, processing, and shipment. We will use an enzyme-linked immunosorbent assay (ELISA) for bioanalytical analysis of secukinumab in serum. The broad principle of the Food and Drug Administration guidance document for “population pharmacokinetics” will be in use.

#### Pharmacogenetics

Additionally, we plan exploratory deoxyribonucleic acid (DNA) research studies to investigate the association between genetic factors and clinical assessments. In particular, these assessments will examine whether individual genetic variation in genes relating to drug metabolism, the indication, and the drug target pathway transmit a differential response to secukinumab. For this purpose, the patients may donate an optional blood sample at baseline. A separate informed consent form is available. As mentioned above, an additional laboratory manual will outline the instructions for sample collection, numbering, processing, and shipment. To maximize confidentiality, all samples and the information associated with the samples will be double-coded to prevent the exposure of the patient’s information and identity. This double-coding process allows the sponsor to go back and destroy the sample at the patient’s request. In case of withdrawal all biological samples not yet analyzed at the time of withdrawal will no longer be used, unless permitted by applicable law. They will be stored according to applicable legal requirement.

##### Sub-studies

The study further includes two optional imaging sub-studies to which the patients can opt-in to participate: ultrasound and contrast-media enhanced MRA to assess vessel wall inflammation before and after treatment with secukinumab. Results from the screening visit will be compared to weeks 28 and 52. An additional imaging assessment in week 60 was added via a third amendment to the protocol to be able to assess vessel inflammation after completed treatment. Patients participating in the trial can be included in the sub studies, if they agree to undergo follow-up examinations. Investigators need to complete a specific training before they can include and follow-up patients in the ultrasound or in the MRA sub-study.

The objectives for the ultrasound sub-study are to determine the number of segments with inflammatory wall thickening (halo sign) of the temporal and axillary arteries before, with, and after treatment with secukinumab versus placebo as well as to measure intima media thickness of each vessel before, with, and after treatment.

If possible, sites should perform the MRA examination in the screening phase prior to any glucocorticoid therapy. To assess the degree of mural inflammation, the contrast-medium images of the arterial wall/perivascular tissue and the wall thickness are evaluated using a 4-point scale (grade 0 = normal to grade 3 = severe inflammation). We consider a wall thickening greater than 0.6 mm with a clearly visible mural contrast-medium uptake an inflammatory sign of the superficial head arteries. The objectives are to examine the presence of an inflammation, a vessel wall thickening, and the amount of contrast-medium uptake before and after treatment with secukinumab.

### Description of outcome measurements

#### Physician’s Global Assessment (PhGA) of disease activity

Physicians will rate the extent to which GCA affects the patients before, with, and after treatment using a 100-mm visual analogue scale [17]. The physician must not be aware of the Patient’s Global Assessment (PGA) in order to ensure objectivity.

#### Patient-reported outcomes

##### Patient’s Global Assessment (PGA) of disease activity

Investigators will ask the patients about the overall effect of GCA on them and globally assess the disease activity by using a 100-mm visual analogue scale (Patient’s Global Assessment visual analogue scale, PGA-VAS) ranging from “has no effect at all” to “worst possible effect” [17].

##### Functional Assessment of Chronic Illness Therapy Fatigue scale (FACIT-Fatigue)

The FACIT-Fatigue scale is a 13-item questionnaire, which assesses the patient-reported fatigue and its impact on daily activities and function [18, 19]. The level of fatigue is measured on a 4-point Likert scale with “4 = not at all fatigued” to “0=very much fatigued”. The purpose of collecting available FACIT-Fatigue© data is to assess the impact of fatigue on patients with GCA.

##### Short Form Health Survey (SF-36)

The SF-36 is a widely used instrument to measure health-related QoL in healthy subjects and patients with acute and chronic conditions. It consists of 8 subscales which can be scored individually: physical functioning, role-physical, bodily pain, general health, vitality, social functioning, role-emotional, and mental health [20]. Each scale is directly transformed into a 0–100 scale; the lower the score the more disability, i.e., a score of “0” is equivalent to maximum disability and a score of “100” is equivalent to no disability.

##### EuroQoL-5D-5L

The EuroQoL-5D-5L (EQ-5D-5L) is a self-administered questionnaire designed to assess health status in adults [21, 22]. It is divided into two sections; the first section includes one item addressing each of 5 dimensions: mobility, self-care, usual activity, pain/discomfort, and anxiety/depression. Patients rate each of these items from “no problem” to “extreme problems/unable”. A composite health index is then defined by combining the levels for each dimension. The result is converted into a point value using a special algorithm. Overall, scores range from “0” to “1” with lower scores presenting lower quality of life.

The second section measures self-rated global health status using a visual analogue scale where “100” represents the “best possible health state” and “0” represents the “worst possible health state”.

#### Laboratory assessments

Prior to study treatment, patients donate a blood sample to examine immunogenicity. Anti-secukinumab antibodies will be determined in serum by Meso Scale Discovery (MSD) assay.

Laboratory will measure hemoglobin, hematocrit, red blood cell count, white blood cell count, and platelet count.

Serum chemistry will include sodium, potassium, blood urea nitrogen/urea, bicarbonate, phosphorous, total protein, calcium, albumin, uric acid, creatinine, creatinine kinase, total bilirubin, aspartate aminotransferase, alanine aminotransferase, gamma glutamyl transferase, and alkaline phosphatase at all study visits.

Fasting blood samples are used to examine lipid profiles including high-density lipoprotein, cholesterol, triglycerides, hemoglobin A1c (HbA1c), and glucose.

CRP and ESR will be determined to identify the presence and severity of inflammation and to monitor the response to treatment.

### Glucocorticoid Toxicity Index (GTI)

GTI v1.0 is used to document the changes in glucocorticoid-associated morbidity (GTI endpoint was shifted to exploratory endpoint section of the study protocol with the third amendment). This index was developed by Miloslavsky et al. [23] as a comprehensive glucocorticoid assessment instrument. The GTI is useful across different disciplines to assess the clinical value of steroid-sparing agents as well as to measure the impact of glucocorticoid toxicity. The GTI consists of the Composite GTI and the Specific List. The Composite GTI reflects toxicity likely to change during a clinical trial. Toxicities vary with glucocorticoid exposure and are weighed and scored. The Specific List is at hand to capture toxicity not included in the Composite GTI. Thirty-one toxicity items are included in the Composite GTI and 23 in the Specific List. Originally, the trial consisted of a 28-week treatment period including final assessments at week 28 after complete prednisolone tapering. Therefore, the sponsor excluded the bone domain and the domain-specific scores from the total GTI since annual bone density measurements are needed. During study conduct, the design was amended and treatment duration was extended up to week 52 to assess the effect of secukinumab after completed steroid tapering with regard to sustained remission, the potential steroid sparing effect, safety, and tolerability up to week 52. A subsequent incorporation of bone density measurements was not purposeful as baseline values were missing.

### Safety assessment/monitoring

The safety analysis includes all randomized patients who received at least one dose of secukinumab. Investigators are supposed to evaluate the occurrence of adverse events (AEs) by non-directive questioning of the patients at each visit. Additionally, the patients may voluntarily inform the investigator of new AEs during/between visits or the investigator may detect AEs through physical examination findings, laboratory test findings, or other assessments. If appropriate, events will be classified as SAEs (serious adverse events), SARs (serious adverse reactions) or SUSARs (suspected unexpected serious adverse reactions).

Site staff needs to monitor ESR and CRP prior to study treatment and at each visit up to end of treatment (refer to Table 1). Patients need to donate samples for hematology, blood chemistry, urinalysis, and lipids (fasted) during the screening phase, at baseline, at weeks 4, 12, 28, 36, 44, and 52. The Sponsor will classify AEs during treatment according to the United States National Cancer Institute, Common Terminology Criteria for Adverse Events version 4.0. Novartis will address AEs with grades 3 to 4 in a timely manner and document them in detail.

Pregnancy is an exclusion criterion. Site personnel needs to perform a serum pregnancy test for all female patients at the screening visit. Further urine pregnancy tests are scheduled at baseline, weeks 3, 12, and 28 for women of childbearing potential.

Patients will be analyzed according to the study treatment received, where treatment received is defined as the treatment the patient received on the first day of study treatment. The objective is to evaluate the safety/tolerability and immunogenicity of secukinumab in patients with newly diagnosed or relapsing GCA. Therefore, safety and tolerability assessments over time are analyzed: incidence and severity of adverse events (AEs) and serious AEs (SAEs). Safety summaries (tables, figures) include only data from the on-treatment period with the exception of baseline data which will also be summarized where appropriate (e.g., change from baseline summaries). All information obtained on AEs will be displayed by treatment group and patient. The number (and percentage) of patients with treatment-emergent AEs will be summarized. Separate summaries will be provided for study medication-related AEs, death, serious AEs, and other significant AEs leading to study drug discontinuation. All vital signs and laboratory data will be listed by treatment group, patient, and visit/time and if ranges are available, abnormalities will be flagged. Summary statistics will be provided by visit and by treatment group and change from baseline will only be summarized for patients with both baseline and post-baseline values.

### Data management and quality control

Sponsor’s personnel will review the data entered by the investigational staff in the electronic case report forms for completeness and accuracy (in line with source data verification). They will create electronic data queries stating the nature of the problem and requesting clarification for discrepancies and missing values and send them to the investigational site via the electronic data capture system. Designated site staff is required to respond promptly to queries and to make any necessary changes to the data. Sponsor’s personnel will document the occurrence of relevant protocol deviations in the mentioned above electronic system.

This study will include a data monitoring committee which will function independently of all other individuals associated with the conduct of this trial, including the site investigators participating in the study. The committee will assess the progress of the trial at defined intervals, safety data, and critical efficacy variables and recommend to the sponsor whether to continue, modify, or terminate the trial.

The final data set will be property of the sponsor. After final database lock, all principal investigators will receive copies of their own site’s data sets.

### Data protection

All investigators and trial staff will comply with the requirements of the German Data Protection Law with regard to collection, storage, processing, and disclosure of the patients’ personal information. The trial evaluation team will receive only anonymized data. Patients’ personal data is stored locally at each study site in locked cabinets or electronically on encrypted secure drives.

### Analysis

#### Statistical analysis

This proof of concept trial will use a Bayesian methodology to obtain posterior distributions for the response rates in both treatment arms. For secukinumab, a non-informative prior- and for placebo an informative prior [based on the responses observed in the GIACTA study [12]] will be used. These priors will be updated to obtain posterior distributions for the response rates and for their difference. Based on the observed response rates in the GIACTA study [12], 56 out of 100 patients (56%) on tocilizumab every other week in combination with 26-week prednisolone taper regimen and 9 out of 51 patients (18%) treated with placebo in combination with 52-week prednisolone taper regimen. The expected observed responders in a sample size of 25 patients per treatment group is then 25 × (56/100) = 14 and 25 × (9/51) = 4. The posterior distribution of the expected difference in proportions was investigated for the sample size of 25 patients per treatment group.

The full analysis set comprises all patients to whom study treatment has been assigned by randomization and who received at least one dose of randomized study treatment (secukinumab or placebo). The risk difference presented with a credible interval estimate of the 95% posterior interval (i.e., the 95% credibility interval), using the 2.5 and 97.5 percentiles as well as the median (50 percentile), will be obtained. The median and 95% credibility interval of the odds ratio (OR), risk difference (RD), and risk ratio (RR) will be presented. In order to assess the robustness of the primary endpoint, the following analyses are planned:

•Using a non-informative prior, i.e., uniform prior Beta (0.5, 0.5), for both treatment groups.

•A logistic regression model with treatment in the model. ORs along with the respective two-sided 80% confidence interval (CI) will be derived for the treatment comparison.

Furthermore, the primary analysis will be repeated based on only signs and symptoms of GCA in order to mitigate against the possibility of biasing.

Patients who do not achieve remission within 12 weeks of baseline, or are in the “escape arm”, or drop out from the study prior to/on week 28, or do not have information to evaluate sustained remission response at week 28, will be classified as non-responders in the primary analysis.

Subgroup analyses of the primary endpoint will be performed to investigate the difference between new-onset (GCA diagnosed within 6 weeks of baseline) and refractory patients (GCA diagnosed ≥ 6 weeks before baseline and previous treatment with ≥ 25 mg/day prednisolone for ≥ 2 consecutive weeks), and to allow assessment of the benefit/risk ratio of secukinumab treatment in patients who need a higher (> 40 mg/day) versus a lower (≤ 40 mg/day) initial dose of prednisone.

The secondary endpoints of this trial will be analyzed as follows:

- The proportion of patients in sustained remission until week 52 (Yes, No) will be summarized descriptively for each treatment group. Note: Sustained remission at week 52 refers to patients without flare until week 52 and in adherence to the protocol prednisolone taper regimen. Flare is determined by the investigator and defined as the recurrence after remission of signs or symptoms of GCA and/or ESR ≥ 30 mm/h and/or CRP ≥ 10 mg/L attributable to GCA. In addition, the reasons why a patient is determined to be a responder or non-responder to patients in sustained remission until week 52 will be summarized.
- The proportion of patients in remission (Yes, No) at each time point will be summarized descriptively for each treatment group. Note: Remission refers to the absence of flare. This summary will only summarize remission information up to 4 weeks post last administration of study treatment.
- The time to first GCA flare after remission (up to and including week 52) will be summarized using Kaplan Meier curves. Descriptive statistics will also be provided for time to first GCA flare after remission in days (up to and including week 52).
- Total cumulative prednisolone dose from the first dose of co-administered treatment to weeks 26, 28, and 52 will be summarized over time by the treatment group.
- The number and percentage of patients on prednisolone dose ≤ 5mg/day at weeks 19, 28, and 52 will be summarized by the treatment group. Note: The prednisolone dose refers to the average co administered treatment dose in the week of interest.
- Descriptive summary statistics for the change as well as relative change from baseline of PhGA VAS to each study visit of interest will be presented for each treatment group. Change from baseline will only be summarized for patients with both baseline and post baseline values.
- Descriptive summary statistics for the change from baseline of CRP (mg/L) and ESR (mm/h) to each study visit of interest will be presented for each treatment group. Change from baseline will only be summarized for patients with both baseline and post baseline values.

The analysis of the PRO secondary endpoints is detailed here:

- Descriptive summary statistics for the change as well as relative change from baseline of PGA VAS to each study visit of interest will be presented for each treatment group. Change from baseline will only be summarized for patients with both baseline and post baseline values.
- Descriptive summary statistics for the change as well as relative change from baseline of FACIT-Fatigue to each study visit of interest will be presented for each treatment group. Change from baseline will only be summarized for patients with both baseline and post baseline values.
- Descriptive summary statistics for the change as well as relative change from baseline of SF 36 domain and summary scores to each study visit of interest will be presented for each treatment group. Change from baseline will only be summarized for patients with both baseline and post baseline values. The proportion of responders at each time point will be summarized descriptively for each treatment group.
- The EQ-5D-5L is a questionnaire with 5 questions (regarding mobility, self-care, usual activities, pain/discomfort, and anxiety/depression) each with 5 categories and a health state assessment from 0 (worst possible health state) to 100 (best possible health state). The number and percentage of patients in each of the 5 categories for each question will be presented by study visit and treatment group. Descriptive summary statistics will be provided for the change from baseline of health state assessment (known as EQ-5D-5L VAS) and EQ-5D-5L utility index by study visit and treatment group. The predicted EQ-5D-5L utility index values range from −0.661 to 1.

AEs will be coded by primary system organ class (SOC) and preferred term (PT) according to the Medical Dictionary for Regulatory Activities (MedDRA) version 22.0 or later and will be presented for the whole study period. We will only summarize treatment emergent AEs, which are events that either emerge during treatment, i.e., AEs which were absent prior to treatment, or events which worsen relative to the pretreatment state.

#### Sample size calculation

We evaluated the planned analysis by simulation techniques. With a sample size of 25 patients per treatment group, >90% of the simulated samples displayed response rates which were similar to those observed in the GiACTA study (12).

## Discussion

The design of this German-wide phase II trial allows us to gain first placebo-controlled data on the efficacy and safety of the IL-17A inhibitor secukinumab in patients with active GCA. The study will explore the hypothesis whether secukinumab has the potential to maintain GCA remission and to reduce glucocorticoid dose/toxicity.

The use of glucocorticoids should be minimized, especially in an older patient population. These patients often have additional comorbidities such as hypertension or diabetes, which can be induced or deteriorate with glucocorticoids. Glucocorticoids also play a major role for developing osteoporosis, especially in postmenopausal women. Bone fractures are common in GCA. Paskins et al. could demonstrate that the fracture risk was higher in patients who received a higher average daily dose of glucocorticoids than in those who received a lower daily dose [24]. Labarca et al. have shown that even after 2 years, only 55% of patients take less than 5 mg prednisolone equivalent [24]. The need for additional glucocorticoid-sparing drugs is high in order to reduce glucocorticoid-induced side effects. It is desirable to reduce the glucocorticoid dose to 5 mg or less prednisolone equivalent as fast as possible. If effective, it can be anticipated from all we know that secukinumab could be a valuable and safe addition to the yet sparse glucocorticoid-sparing therapy repertoire for GCA.

The precise recognition of relapses is one of the most challenging aspects in GCA treatment. ESR and CRP are useful adjuncts to clinical decision making [25]. Increased levels of inflammation parameters are almost always present. IL-6 is the major mediator for the hepatocytic secretion of most of the acute phase proteins, including CRP [26]. IL-6 receptor blockers are known to directly suppress acute phase reactants which are essential to currently applied remission and relapse criteria [3, 27–29]. Especially in older patients with GCA, CRP monitoring plays a decisive role in the assessment of progression and relapses. Inflammatory markers are particularly important for follow-up in patients who cannot adequately express their symptoms, e.g., patients with dementia. Further, symptoms arising from concomitant disease, such as pain in shoulder or hip osteoarthritis, may be overinterpreted. In comparison to tocilizumab, secukinumab does not directly suppress acute phase proteins. The antibody has an indirect anti-inflammatory impact on acute phase proteins by inhibiting IL-17A, which can induce, e.g., IL-6 production, which can, as outlined above, promote myeloid-driven innate inflammation, including CRP.

In order to assess the true benefit of new medications with regard to their glucocorticoid-sparing properties, investigators must be able to assess their ability to prevent or reverse glucocorticoid-related adverse events [23]. To our knowledge, this study is the first that uses the GTI to examine the potential positive outcomes with secukinumab. Miloslavsky et al. developed the GTI, a comprehensive instrument for the assessment of glucocorticoid toxicity.

An interesting feature of the study is the application of imaging techniques (MRA, ultrasound). In comparison to temporal artery biopsies, ultrasound and MRA are safe, less invasive, and well tolerated by the patients. Results are quickly available, so that physicians can initiate a necessary therapy immediately with higher diagnostic safety. A EULAR (European League Against Rheumatism) task force recommends that the diagnosis of GCA can be confirmed by imaging (MRA, ultrasound, computerized tomography or positron emission tomography) alternatively to histology from temporal artery biopsy. Imaging is less invasive, results are immediately available, and sensitivity is higher as more arteries can be investigated. Furthermore, imaging has the potential for follow-up investigations, therefore, contributing to a lower number of false negative results [30]. The use of these imaging techniques at baseline and during follow-up should show a potential effect of the treatment with secukinumab on vessel wall thickness and intraluminal diameters.

Ultrasound of temporal and axillary arteries has the potential as an outcome instrument. It shows a non-compressible, most commonly concentric thickening of the vessel wall (halo-sign, compression sign) in acute disease [31–34]. A recent meta-analysis of prospective studies has shown a pooled sensitivity of 77% and a pooled specificity of 96% for temporal artery ultrasound when compared to the final clinical diagnosis of GCA. The positive and negative likelihood ratios are 19 and 0.2, respectively [35]. Reliabilities of sonographers reading videos are comparable to reliabilities of pathologists reading temporal artery biopsy specimen [36]. The EULAR Recommendations for Imaging in Large Vessel Vasculitis suggest ultrasound of temporal/axillary arteries as the first imaging modality particularly in patients with suspected cranial GCA [30]. Several publications demonstrated that the halo-sign disappears in the temporal arteries within the first three weeks of treatment in the majority of patients while it resolves only within months or years in the axillary arteries [32, 37–43].

MRA enables the detection of soft tissue swelling of the wall of large arteries and the aorta and provides information about the luminal anatomy and blood flow. Thus, MRA is helpful for detecting GCA-related vascular stenosis or aneurysms [3]. A systematic literature review by Duftner et al. revealed a good performance of magnetic resonance imaging (MRI) for the diagnosis of cranial GCA with a pooled sensitivity of 73% and a pooled specificity of 88% [35]. A EULAR recommendation states that physicians should consider a high-resolution MRI of superficial cranial arteries, if ultrasound is not available or inconclusive [30]. The two sub-studies should give a first hint to the question whether secukinumab in combination with glucocorticoids possibly leads to stronger reduction of vascular wall inflammation than a glucocorticoid monotherapy.

The study has limitations: it has only a modest sample size. However, we assume that these results may give a first idea whether it is worthwhile to initiate investigations that are more extensive. Successful achievement of the primary endpoint could support the continuation of clinical activities in GCA research.

## Trial status

The valid trial protocol at the time of submission is version 03, dated 29 November 2019.

Revision chronology: First amendment released on 15 March 2019, second amendment on 13 May 2019, and third amendment on 29 November 2019.

Recruitment started in Q1/2019 with first patient enrollment on 30 January 2019. The study completed recruitment in April 2020, 5 months ahead of schedule, and hence, the estimated trial completion date was shifted from Q4/2021 to Q3/2021.

## Data Availability

Not applicable.

## Appendix

### Rationale for using IL-17A as therapeutic target

In GCA, pathomechanisms triggering and maintaining the inflammatory cascade are incompletely understood. Several advances in genetic and immunology research have provided a greater understanding of the pathogenesis of GCA, resulting in new potential therapeutic targets.

During the initial phase of GCA, dendritic cells in the adventitia of normal arteries are activated. Subsequently, these activated dendritic cells induce the proliferation and polarization of interferon-gamma producing Th1 and IL-17 producing Th17 cells, respectively [44]. Inflammation-related activation and proliferation of the vascular intima may result in critical narrowing or even occlusion of the vascular lumen with the risk of post stenotic ischemia potentially leading to irreversible tissue destruction [45]. IL-17A seems to play a role in the pathogenesis of GCA; Espígol-Frigolé et al. found that IL-17A expression in temporal artery lesions is increased and is a predictor of sustained response to glucocorticoid treatment in patients with GCA. While IL-17A was remarkably upregulated in lesions, it was barely detectable in serum. The authors did not find a relationship between IL-17A expression and systemic symptoms or acute-phase proteins. They assumed that IL-17A functions are predominantly exerted locally at the vascular lesions [46, 47]. Márquez et al. found a novel association between polymorphisms within the IL-17A locus and GCA that supports the relevant role of Th17 cells in pathophysiology of GCA [48]. A study by Miyabe et al. showed hyperproliferation of regulatory T-cells that overexpress a hypofunctional isoform of FoxP3 that lacks exon 2 (FoxP3Δ2). The dysfunctional FoxP3Δ2 domain contributes to an enhanced Th17 differentiation and, therefore, IL-17A overproduction. In line with this observation, IL-17A was upregulated in active GCA patients in this study. The IL-6 receptor antagonist tocilizumab is able to re-establish the functional Foxp3 domain in regulatory T-cells leading to less IL-17A production and effective control of GCA [49]. Additionally, two case reports of patients with both GCA and psoriatic arthritis describe a remission on treatment with secukinumab: Rotar et al. [50] report the first case of remission maintenance in GCA with secukinumab. Here, in a 67-year-old female patient psoriatic arthritis flared. Treatment with tocilizumab and leflunomide was stopped and secukinumab was started. The loading dose was 300 mg subcutaneously weekly for 5 weeks and therapy was continued with 300 mg subcutaneously every 4 weeks. In parallel, treatment with methylprednisolone was tapered and discontinued. One year after the last tocilizumab infusion, the patient had no symptoms of GCA or psoriatic arthritis (clinical disease activity index 0, erythrocyte sedimentation rate [ESR] 28 mm/h, C-reactive protein [CRP] <5 mg/L). In a 70-year-old male patient reported by Sammut et al. [51], psoriatic arthritis was managed with adalimumab. After diagnosis of GCA therapy with oral prednisolone was initiated. The patient remained on adalimumab but psoriatic arthritis flared and glucocorticoid requirement for GCA remained high. Three months later, therapy with adalimumab was stopped and secukinumab was started. Four months later, both psoriatic arthritis and GCA were in remission and CRP had normalized on low dose glucocorticoid. Nine months after presentation with GCA, the patient remained well on low dose glucocorticoid and secukinumab.

All of these observations promote the idea of IL-17A inhibition as a potential treatment target in GCA.

### Rationale for the choice of glucocorticoid combination therapy

High-dose glucocorticoid therapy is the mainstay of treatment for GCA. Yet, this therapy is associated with serious disadvantages for the patients. Therefore, a treatment that would reduce the dose and duration of glucocorticoid therapy would be of great benefit. In our study, a taper regimen was selected which has already been applied in combination with another humanized monoclonal antibody (tocilizumab) in the GiACTA trial [12]. In this phase III trial, the percentage of placebo patients in remission following the 26-week taper regimen versus the 52-week taper regimen was comparable [14% versus 18% [12]]. The incidence of adverse events (AEs)/SAEs was numerically higher in the placebo groups than in the tocilizumab groups, with the highest one in the 52-week taper regimen placebo group. This may have been the result of the effects of glucocorticoids. In the 26-week taper regimen group, no ischemic event and in particular no cases of acute visual impairment due to anterior ischemic optic neuropathy or other GCA-related causes were reported [12]. Due to the glucocorticoid-therapy associated AEs and their substantial increase during long-term use, efforts should be made to minimize glucocorticoid dose and duration when treating patients with GCA [52]. Thus, the 26-week glucocorticoid (i.e., prednisolone) taper regimen, with a low-stringency escape strategy (i.e., escape treatment with prednisolone is allowed for any patient with persistent active disease at 12 weeks or flare following remission), was used for this study.

## Abbreviations

AE: Adverse event
CRP: C-reactive protein
DNA: Deoxyribonucleic acid
ELISA: Enzyme-linked immunosorbent assay
ESR: Erythrocyte sedimentation rate
EULAR: European League against Rheumatism
FACIT: Functional assessment of chronic illness therapy
GCA: Giant cell arteritis
GTI: Glucocorticoid toxicity index
IL: Interleukin
MedDRA: Medical Dictionary for Regulatory Activities
MRA: Magnetic resonance angiography
MRI: Magnetic resonance imaging
MSD: Meso Scale Discovery
PGA: Patient’s Global Assessment
PhGA: Physician’s Global Assessment
PMR: Polymyalgia rheumatica
PT: Preferred term
QoL: Quality of life
SAE: Serious adverse event
s.c.: Subcutaneously
SOC: System organ class
Th: T helper cells
TNF: Tumor necrosis factor
ULN: Upper limit of normal
VAS: Visual analogue scale
